# Pamidronate-Conjugated Biodegradable Branched Copolyester Carriers: Synthesis and Characterization

**DOI:** 10.3390/molecules22071063

**Published:** 2017-06-26

**Authors:** Ewa Oledzka, Dagmara Pachowska, Katarzyna Orłowska, Joanna Kolmas, Agata Drobniewska, Ramona Figat, Marcin Sobczak

**Affiliations:** 1 Department of Biomaterials Chemistry, Chair of Inorganic and Analytical Chemistry, Medical University of Warsaw, Faculty of Pharmacy with the Laboratory Medicine Division, Banacha 1, 02-097 Warsaw, Poland; dagmara.pach@interia.pl (D.P.); katarzyna.orlowska136@gmail.com (K.O.); marcin.sobczak@wp.pl (M.S.); 2 Department of Inorganic and Analytical Chemistry, Chair of Inorganic and Analytical Chemistry, Medical University of Warsaw, Faculty of Pharmacy with the Laboratory Medicine Division, Banacha 1, 02-097 Warsaw, Poland; joanna.kolmas@wum.edu.pl; 3 Department of Environmental Health Science, Medical University of Warsaw, Faculty of Pharmacy with the Laboratory Medicine Division, Banacha 1, 02-097 Warsaw, Poland; agata.drobniewska@wum.edu.pl (A.D.); ramona.figat@wum.edu.pl (R.F.)

**Keywords:** hydroxyapatite, composite, polymer conjugate, selenium, polyester carrier, controlled release rate, drug delivery systems

## Abstract

The need for development of comprehensive therapeutic systems, (e.g., polymer-apatite composites) as a bone substitute material has previously been highlighted in many scientific reports. The aim of this study was to develop a new multifunctional composite based on hydroxyapatite porous granules doped with selenite ions (SeO_3_^2−^) and a biodegradable branched copolymer-bisphosphonate conjugate as a promising bone substitute material for patients with bone tumours or bone metastasis. A series of biodegradable and branched copolymer matrices, adequate for delivery of bisphosphonate in the bone-deficient area were synthesized and physico-chemically and biologically (cyto- and genotoxicity assays) characterized. Branched copolymers were obtained using a hyperbranched bis-MPA polyester-16-hydroxyl initiator and Sn(Oct)_2_, a (co)catalyst of the ring-opening polymerization (ROP) of l,l-lactide (LLA) and ε-caprolactone (CL). A new amide bond was formed between the hydroxyl end groups of the synthesized copolymer carriers and an amine group of pamidronate (PAM)—the drug inhibiting bone resorption and osteoclast activity in bone. The dependence of the physico-chemical properties of the copolymer matrices on the kinetic release of PAM from the synthesized branched copolymer conjugate-coated hydroxyapatite granules doped with selenite ions was observed. Moreover, the correlation of these results with the hydrolytic degradation data of the synthesized matrices was evidenced. Therefore, the developed composite porous hydroxyapatite doped with SeO_3_^2−^ ions/biodegradable copolymer-PAM conjugate appears most attractive as a bone substitute material for cancer patients.

## 1. Introduction

Polymer conjugates are a particular type of product in which a drug is covalently conjugated to a carrier, usually a macromolecular substance (polymer, protein or DNA) [1]. Their task is to deliver a drug to a specific site of action in order to improve its properties and increase the efficiency of treatment. The application of polymer conjugates is associated with the reduction of drug side effects and toxicity, as well as improving bioavailability and pharmacokinetics [2].

Disodium pamidronate (PAM) belongs to the second generation of bisphosphonates. PAM blocks the access of osteoclast precursors to the bone and inhibits the activity of osteoclasts, thereby reducing bone resorption. Furthermore, it inhibits the formation and dissolution of hydroxyapatite crystals when it is used in vitro. The application of PAM has resulted in hypercalcaemia reduction and a decrease in the excretion of bone resorption products in urine [3]. PAM is poorly absorbed from the gastrointestinal tract and hence is rarely used in this form. It is mainly administered intravenously. PAM is bound to plasma proteins in approximately 50%, and 20–55% of the dose applied is excreted in the urine within 72 h, while the rest of the drug penetrates to the bone. It is not metabolized, and its half-life in bone is about 300 days [4]. The application of PAM is indicated for the treatment of disorders associated with an increase in osteoclast activity, in particular of bone tumours or Paget’s disease. Moreover, PAM is the preferred bisphosphonate (together with zoledronic acid) for the treatment of severe hypercalcemia.

The development of the polymer-bisphosphonate conjugates has become particularly important due to the low drug bioavailability and the occurrence of adverse effects after its application. Recently, new biomaterials of this type were synthesized and physico-chemically characterized, namely, conjugates of poly(lactide-*co*-glycolide) (PLGA) with the bisphosphonate drug alendronate (PLGA-ALE) [5], polyethylene glycol (PEG)-conjugated alendronate [6], and alendronate-conjugated amphiphilic hyperbranched polymer based on Boltorn H40 and PEG [7]. Furthermore, pamidronate-polymer conjugates were prepared using pullulan as a carrier attached to the drug via an amide bond [8]. The conjugation reactions were carried out in the presence of *N*,*N'*-carbonyldiimidazole (CDI). The application of a fluorescent probe allowed the use of the resulting conjugates for imaging and diagnosis of the bone tissue regeneration. Magnetic resonance imaging (MRI) studies in mice, however, have confirmed the effectiveness of the prepared biomaterials. In addition, the polymer-bisphosphonate conjugates are often used as elements of the hydroxyapatite composite. The development of such a spatial frame allows for its implementation into bone defects. Recently, the production of such multifunctional composites for application as a bone graft substitute with the proper mechanical and pharmacological properties was carried out [9,10]. Typically, such biomaterials comprise three components: the active hydroxyapatite, natural or synthetic polymer or copolymer and a drug (or growth factor) [9,10].

The biodegradability, biocompatibility and permeability of the aliphatic polyesters, (e.g., poly(l-lactide) (PLA), poly(l,l-lactide) (PLLA), poly(ε-caprolactone) (PCL) and the copolymers of d,l-lactide (LA), l,l-lactide (LLA) and ε-caprolactone (CL)) make them suitable materials for the creation of variety of implantable and/or controlled-injection systems for drug release [11,12]. These materials are usually synthesized by the ring-opening polymerization (ROP) of proper monomers in the presence of multifunctional initiators containing nucleophile groups (hydroxyl or amine) [13].

Hydroxyapatite (HAp) is based on calcium phosphate, which is similar to human hard tissues in morphology and composition [14]. HAp has found a wide application in medicine, where it has been successfully used as an implantation material, due to its high biocompatibility, porosity and high corrosion resistance [15]. HAp plays an important role in preventive, restorative and regenerative dentistry. It is a component of toothpastes and dental cements and fillers. HAp is widely used to reconstruct periodontal bone defects and, as a coating of dental implant, to improve osteointegration. Moreover, HAp in different 3D forms may be apply in maxillofacial surgery as a bone substitute [16].

Selenium is a trace element essential to the proper functioning of living organisms. It is a constituent of many proteins, known as selenoproteins. However, its most important role is to protect cells against oxidative stress. Selenium is a constituent of glutathione peroxidase, which is responsible for detoxification of free radicals and peroxides [17]. This element is also considered to be an anticarcinogenic factor. Furthermore, selenium reduces the risk of vascular diseases and heart attacks, stimulates the immune system and plays an important role in transmitting nerve signals [18]. Many scientific reports indicate the need for developing comprehensive therapeutic systems, (e.g., polymer-apatite composites) for increasing patient comfort. The resulting composites could be used as a bone substitute material for cancer patients. Additionally, bisphosphonates, (e.g., PAM), covalently conjugated to a biodegradable polymer carrier, increase the efficacy of treatment by acting directly on the application site.

Therefore, the main aim of our study was to develop a new multifunctional composite based on hydroxyapatite granules doped with selenite ions (SeO_3_^2−^) and a biodegradable branched copolymer-PAM conjugate, as a promising bone substitute material for patients with bone tumours or bone metastasis (jawbone). To the best of our knowledge, no research group has previously reported on the synthesis and characterization of such biomedical composites. In the present work, we demonstrate the synthesis, characterization and drug-release behaviour of a newly formed conjugate of PAM that is bonded to biodegradable branched matrices. To achieve this goal, the copolymers were firstly obtained by ROP of LLA and CL using a poly-2,2-bis(methylol)propionic acid (bis-MPA) initiator. The synthesized copolymeric matrices were subjected to cyto- and genotoxicity assays and then bonded to PAM. The in vitro release rate of the drug, as released from the synthesized conjugates coating the porous hydroxyapatite granules doped with selenite ions, was analysed.

## 2. Results

In bone regeneration, two methods for the local administration of bisphosphonate have been previously investigated [19]. One method is to use the bisphosphonate to immobilize implants in order to enhance bone quality around the implant, and the second method is to apply a drug delivery system (DDS) in order to deliver the drug to the bone-deficient area [19]. Therefore, in this work, the efficiency of porous hydroxyapatite composite doped with selenite ions and coated with PAM-branched copolymer conjugate, for local drug administration, was investigated.

### 2.1. Copolymeric Branched Matrices Synthesis and Characterization

Biodegradable and branched copolymer matrices suitable for delivery of bisphosphonate in the bone deficient area were designed and synthesized. Branched bis-MPA-PLLA-PCL copolymers were obtained using hyperbranched bis-MPA polyester-16-hydroxyl initiator and Sn(Oct)_2_, a (co)catalyst of the ROP of LLA and CL. The copolyesters with three different molar ratios were synthesized in a two-step polymerization process (Table 1). First, the macroinitiator (bis-MPA-LLA) was synthesized by adding LLA to bis-MPA. The copolymers were completed by adding of a CL monomer to react further with the bis-MPA-LLA macromolecule.

**Figure 1:**
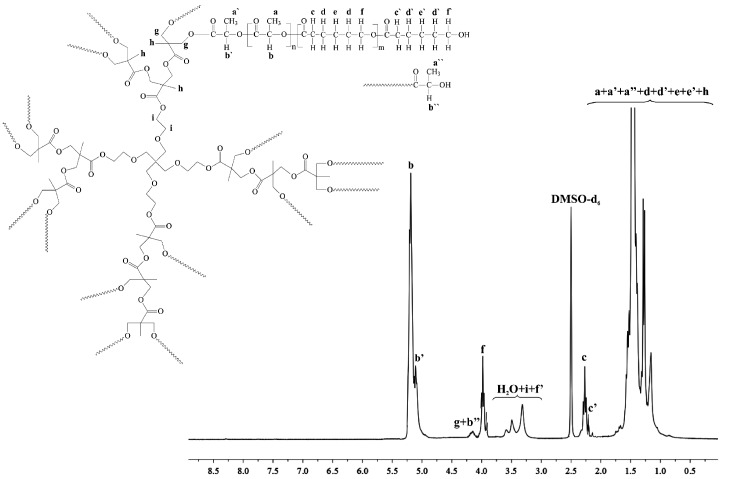
^1^H-NMR spectrum of bis-MPA-PLLA90-PKL38 sample.

The molecular weights of the synthesized branched copolymers were in the range of 18,800–21,000 Da with polydispersity ranging from 1.5 to 1.6. In Table 1, the average degree of polymerization (*DP*) and average degree of substitution (*DS*) of the obtained branched copolymers were determined. The results appear that between 10 and 13 copolymer arms can be attached to the surface of bis-MPA initiator. It was noted that there are unreacted hydroxyl groups in the bis-MPA molecule. This fact may be attributed to the change in the density and distribution of hydroxyl groups on the surface of the initiator and the stearic hindrance of the attached growing copolymer chains [20]. However, it was difficult to find and discriminate unreacted hydroxyl groups of bis-MPA moiety on ^1^H-NMR spectra. The synthesized branched copolymers with bis-MPA core were characterized by ^1^H-, ^13^C-NMR and FT-IR analysis (Figure 1, and Figures A and B, Supplementary Materials). Figure 1 shows the ^1^H-NMR spectra of bis-MPA-PLLA90-PKL38 copolymeric carrier.

The major resonance signals assigned to methylene or methine proton signals terminated by hydroxyl end groups and also PCL and PLLA chains may be easily observed in Figure 1. It is crucial to note that the characteristic proton signals of bis-MPA initiator (assigned as i, h, g) were also detected with high resolution, indicating incorporation of bis-MPA into macromolecule. ^1^H-NMR analysis demonstrated that the initiator used effectively initiated ROP of LLA to form bis-MPA/PLLA macroinitiator and then biodegradable bis-MPA-PLLA-PCL copolymers. An additional confirmation of the presence of bis-MPA initiator in the macromolecule is given by ^13^C-NMR and FT-IR spectra (Figures A and B, Supplementary Materials).

Materials applied in the medical and pharmaceutical areas must meet the relevant requirements (e.g., toxicity effect (PE) should be lower than 20) [21], thus the synthesized branched copolymeric matrices were subjected to cyto- and genotoxicity assays. The luminescent bacteria *Vibrio fischeri* and ciliated protozoan *Spirostomum ambiguum* as well as bacteria *Salmonella typhimurium* were used to perform toxicity tests. It has been found that in the Spirotox test all the extracts were non-toxic (Table 2). Microtox assays were subjected to extraction at a concentration of 90%, which corresponds to the copolymer concentration of 0.9 mg/mL. Table 2 shows the average results for two incubation times of 15 and 30 min. For all copolymeric extracts, the effect was lower than 20%. This occurrence provides information about non-toxicity of the synthesized matrices for luminescent bacteria. In the *umu*-test, it was found that none of the tested samples had been toxic to *S. typhimurium* (Table 3). Compared to a negative control, no growth inhibition was observed. None of the tested samples showed genotoxic potential in both, metabolic activation and non-activation variant (IR < 1.5).

### 2.2. In vitro PAM Release Characteristic and the Branched Copolymer Conjugates Synthesis

**Scheme 1:**
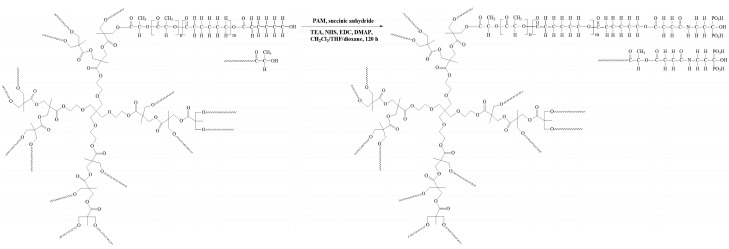
Schematic outline for the synthesis of the branched PAM conjugates.

In the present study, we have conjugated the –OH end group of branched copolymeric chains of the bis-MPA-PLLA64-PCL64, bis-MPA-PLLA90-PCL38 and bis-MPA-PLLA38-PCL90 carriers to the –NH_2_ group of PAM to form a stable branched copolymer-drug conjugates (Scheme 1).

**Figure 2:**
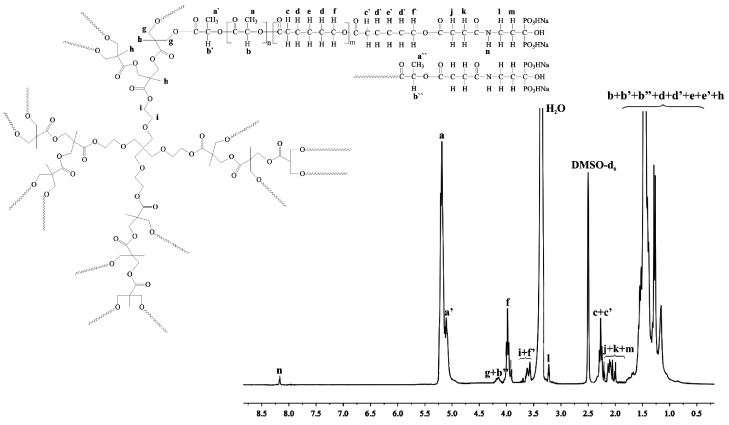
^1^H-NMR spectrum of bis-MPA-PLLA90-PKL38-PAM conjugate.

The formation of the conjugates such as bis-MPA-PLLA64-PCL64-PAM, bis-MPA-PLLA90-PCL38-PAM and bis-MPA-PLLA38-PCL90-PAM was proved by ^1^H-NMR spectroscopy that showed the proton resonance signals corresponding to both, the parent drug and branched copolymeric carrier (Figure 2).

The presence of multiple proton resonance signals of PAM (l, m) together with the distinctive i, h**,** g protons of bis-MPA initiator and the characteristic a, b, c, d, e, f signals of PLLA and PCL confirmed the formation of the branched bis-MPA-PLLA-PCL-PAM conjugates. Similarly, apart from the above characteristic signals, the proton resonance at 8.22 ppm (n, Figure 2) that corresponded to the newly formed -NH group verified the effective synthesis of the intended branched conjugates.

**Figure 3:**
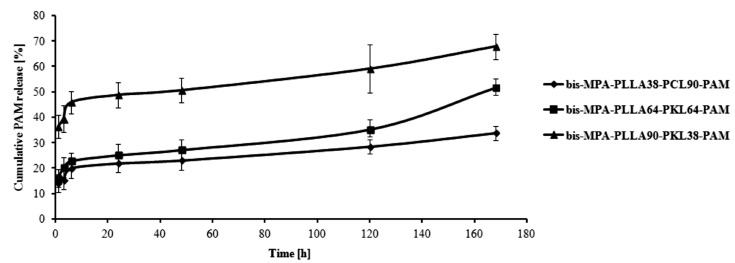
Release profile of PAM from the synthesized branched conjugates.

The fabricated porous hydroxyapatite granules doped with selenite ions were then coated with the synthesized conjugates for the formation of multifunctional composite for the application as a bone substitute material for patients with bone tumour or bone metastasis. In vitro release characteristic of PAM after 7 days of incubation at pH = 7.4 is presented in Figure 3.

As it could easily be observed, the largest amount of PAM was released from the bis-MPA-PLLA90-PKL38-PAM sample, namely 37% and 46% of the drug was released after 1 and 6 hours of incubation. From this point on, could be considered that up to 48 h, a plateau period occurred, where the percentage of PAM released did not exceed 48%. After that, the amount of PAM released increased to finally reach 66% after 168 h of incubation (Figure 3). In the case of bis-MPA-PLLA64-PKL64-PAM conjugate, 16% of PAM was released after 1 h of incubation and almost 22% after 6 h. The total percentage of PAM released (50%) was achieved after 168 h of incubation for this sample. However, only 31% of PAM was released at the same time for the last sample—bis-MPA-PLLA38-PKL90-PAM conjugate. Nonetheless, it should be noted that for this conjugate, the drug was released in the most uniform and controlled manner (Figure 3).

**Figure 4:**
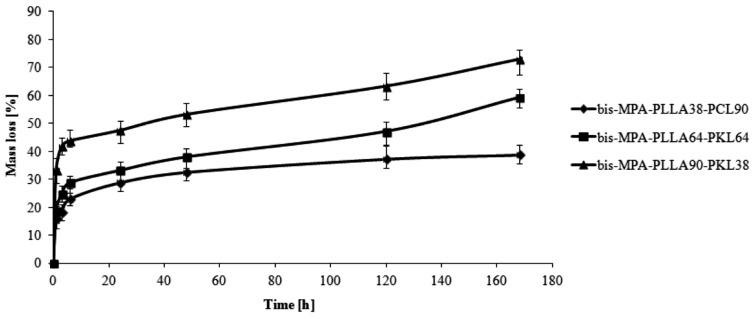
Effect of hydrolytic degradation time on the WL of the branched copolymeric matrices.

As presented in Figure 3, three synthesized copolymeric conjugates show different release profiles, probably due to their various physicochemical properties resulting from different molar ratio of LLA and CL in the macromolecule. PLLA is a hydrophilic polymer and could easily degrade, therefore almost 70% of the drug was released from the bis-MPA-PLLA90-PKL38-PAM conjugate during incubation. Contrary to this, a greater proportion of hydrophobic and crystalline PCL in the macromolecule (bis-MPA-PLLA38-PKL90-PAM sample) resulted in almost three times less amounts of PAM released after 168 h of incubation. The drug release characteristic was closely correlated with the hydrolytic degradation results of the synthesized matrices by monitoring their WL as is shown in Figure 4.

The profile of PAM hydrogel composites was carried out by Aderibigbe and Ndwabu [22]. They demonstrated that the release behaviour of the hydrogels was in agreement with their water sorption capacity. At pH 1.2, between 17% and 27% of PAM was released over a period of 72 h and at pH 7.4, between 56% and 84% of PAM was released over a period of 24 h. At pH 7.4, the release exponent was between 1.03 and 1.39 suggesting super case, 2 whereas at pH 1.2, the release exponent was less than 0.5 indicating quasi-Fickian diffusion. PAM containing implants prepared from spray-dried microparticles were investigated using a laboratory ram extruder at Kissel and coworkers work [23]. PAM-containing polymer matrix consisting of PAM-chitosan implant embedded in the biodegradable polymer d,l-poly(lactide-*co*-glycolide acid-glucose) (PLG-GLU) was compared with a matrix system with the micronized drug distributed in the PLG-GLU. They found that the PAM–chitosan matrix system showed a triphasic release behaviour at loading levels of 6.9 and 15.5% (*w*/*w*) over 36 days under in-vitro conditions. At higher loading (31.9%), a drug burst was observed within 6 days due to the formation of pores and channels in the polymeric matrix. In contrast, implants containing the micronized drug showed a more continuous release profile over 48 days up to a loading of 31.8% (*w*/*w*). At a drug loading of 46.2% (*w*/*w*), a drug burst was observed [23]. Furthermore, PAM control release using hydroxyapatite granules was investigated by Yoshinari and coworkers [19]. The authors prepared different PAM-HAp composites and measured the concentration of PAM released from these composites. They found that the concentration of PAM released could be controlled by regulating the sintering temperature of HAp as a carrier [19].

Developing a new drug form that could potentially improve the drug’s pharmacokinetics as well as simultaneously reducing its adverse effects was the main purpose of this work. PAM has a strong affinity for HAp. Coating the HAp granules doped with selenite ions with the PAM-biodegradable and branched copolymeric conjugate could allow the drug’s therapeutic benefit to be increased since its pharmacokinetics might be improved by the hydrolysis of the newly-formed amide bond. In addition, a potential cancer therapy could be assisted by the presence of selenite ions in the manufactured composite. Furthermore, there is a possibility of developing a therapeutic system that can be applied directly to the bone cavity, thus creating opportunities for targeted therapy. Such systems would be extremely useful for the treatment of bone cancers, bone metastases or osteoporosis.

The HAp application in the development of such a composite improves its physical properties and extends its applicability; it increases its durability, reduces degradation time and significantly increases biocompatibility. Importantly, the synthesized novel bisphosphonate conjugate can also be used to coat other ceramic or metallic materials in order to increase their biocompatibility as well as to provide appropriate therapeutic properties.

## 3. Materials and Methods

### 3.1. General Information

Disodium pamidronate hydrate (PAM, purity >97.0%, TCI EUROPE N.V., Zwijndrecht, Belgium) was dried before use. Succinic anhydride (97.0%, Aldrich Co., Poznan, Poland), *N*-hydroxysuccinimide (98.0%, Aldrich Co., Poznan, Poland), *N*-(3-dimethylaminopropyl)-*N*′-ethylcarbodiimide hydrochloride (EDC, 98.0%, Aldrich Co., Poznan, Poland), triethylamine (TEA, ≥99.0%, Sigma-Aldrich Co., Poznan, Poland) and 4-(dimethylamino)pyridine (DMAP, ≥99.0%, Aldrich Co., Poznan, Poland) were used without further purification. ε-Caprolactone (CL, 2-oxepanone, ≥99.0%, Aldrich Co., Poznan, Poland) was dried and distilled before use over CaH_2_ at reduced pressure. l,l-Lactide (LLA, *cis*-3,6-Dimethyl-1,4-dioxane-2,5-dione, 98.0%, Aldrich Co., Poznan, Poland) was recrystallized from dried ethyl acetate in a dry nitrogen atmosphere and then thoroughly dried in vacuum before use. Hyperbranched bis-MPA polyester-16-hydroxyl, generation 2 (bis-MPA, ≥97.0%, Aldrich Co., Poznan, Poland) were thoroughly dried in a vacuum. Dichloromethane (anhydrous, ≥99.8%, Avantor Performance Materials Poland S.A., Gliwice, Poland), dioxane (anhydrous, 99.8%, Avantor Performance Materials Poland S.A., Gliwice, Poland), THF (anhydrous, 99.8%, Avantor Performance Materials Poland S.A., Gliwice, Poland) and diethyl ether (anhydrous, 99.8%, Avantor Performance Materials Poland S.A., Gliwice, Poland) were used as received. Phosphate buffer solution (pH 7.4 ± 0.05, 0.1 M (potassium dihydrogen phosphate/disodium hydrogen phosphate, 20 °C, Avantor Performance Materials Poland S.A., Gliwice, Poland) was also used as received.

The polymerization products were characterized in DMSO-*d*_6_ solution by means of ^1^H- (300 MHz) and ^13^C-NMR (75 MHz) spectroscopy (Varian, LabX, Midland, ON, Canada). FT-IR spectra of KBr pellets were measured in the 400–4000 cm^−^^1^ range using a Spectrum 1000 spectrometer (Perkin Elmer, Rodgau, Germany).

Number-average molar masses (*M*_n_) and polydispersity indexes (*M*_w_/*M*_n_) of the obtained polymeric products were determined using an Agilent 1100 isocratic pump (GenTech Scientific, Arcade, NY, USA) and a set of two PLgel 5 m mixed-C columns (Agilent, Santa Clara, CA, USA). An Optilab rEX Wyatt interferometric refractometer and a DOWN EOS Wyatt (Wyatt Technology Corporation, Santa Barbara, CA, USA) laser photometer were applied as detectors in a series. ASTRA 4.90.07 software (Wyatt Technology Corporation) was used for data collection and processing. Dichloromethane was used as an eluent at a flow rate of 0.8 mL min^−^^1^ and polystyrene as a standard.

The quantity of pamidronate released was analysed by means of HPLC CAD using an UHPLC Ultimate 3000 analytical system (Dionex, LabX, Midland, ON, Canada) equipped with a CAD detector. Chromatographic separations were carried out using a Luna C8 column (250 × 4.6 mm, 5 μm, Phenomenex Inc., Torrance, CA, USA). The calibration curve was obtained by the analysis of different concentrations of PAM in PBS solution (0.15–2.00 mg/mL). The analytical method was validated by the Pharmaceutical Research Institute in Poland.

### 3.2. Synthesis of the Branched Copolymeric Carriers

The copolymeric materials were synthesized using different molar ratios of initiator (bis-MPA) to co-monomers (l,l-lactide (LLA) or ε-caprolactone (CL)). The initiator/co-monomers feed ratios for the obtained copolymers were: 1:64:64; 1:90:38 and 1:38:90 (mol/mol) denoted as bis-MPA-PLLA64-PCL64, bis-MPA-PLLA90-PCL38 and bis-MPA-PLLA38-PCL90 respectively, where bis-MPA = hyperbranched bis-MPA polyester-16-hydroxyl; PLLA = poly(l,l-lactide) and PCL = poly(ε-caprolactone). For each polymerization, dry bis-MPA and LLA were precisely weighted and introduced into 100 mL polymerization tubes. The tube was then connected to a Schlenk line, where exhausting-refilling processes were repeated three times and then immersed in an oil bath at 130 °C under an argon atmosphere for 48 h. After an appropriate time, the precise amount of CL and a catalytic amount of Sn(Oct)_2_ (0.02 mol % relative to the total monomer concentration) were added to the mixture and the exhausting-refilling process was carried out again (110 °C, 24 h). The obtained copolymers were dissolved in a dry CH_2_Cl_2_, precipitated twice from cold diethyl ether and dried in a vacuum for 72 h.

### 3.3. Spectral Characterization of Copolymeric Biodegradable Matrices (bis-MPA-PLLA90-PKL38 (^1^H-NMR and FT-IR) and bis-MPA-PLLA38-PCL90 (^13^C-NMR) as Examples)

^1^H-NMR (DMSO-*d*_6_, 300 MHz, *δ*_H_, ppm); 5.13 (q, -CH(CH_3_)- of PLLA (a)), 5.07 (q, -CH(CH_3_)- of PLLA (a′)), 4.12 (m, -C(O)C(CH_3_)CH_2_O of bis-MPA and (q, -CH(CH_3_), end group of PLLA (g + b′′), 3.98 (t, -CH_2_O- of PCL (f)), 3.55 (t, -CH_2_OH, end group of PCL (f′)), 3.39 (m, -OCH_2_CH_2_O- of bis-MPA (i)), 2.23–2.21 (t, -CH_2_CH_2_C(O)- of PCL (c + c′)), 1.62–1.22 (m, -CH_2_CH_2_C(O)- of PCL (d + d′) and (m, -CH_2_CH_2_CH_2_- of PCL (e + e′) as well as (d, -CH_3_ of PLLA (a + a′)), (d, -CH_3_, end group of PLLA (a′′), 1.19–1.01 (m, CH**_3_** groups of bis-MPA (h) (Figure 1).

^13^C-NMR (DMSO-*d*_6_, 300 MHz, *δ*_H_, ppm); 174.5–173.91 (a′ + j), 173.17 (m), 172.14 (m′), 169.45 (a + a′′), 68.25 (b + b′′), 67.51 (b′), 64.31 (g′), 63.22 (g + l), 60.98 (g′′), 51.75 (h), 49.64 (i), 33.22 (d), 33.01 (d′), 27.72–23.98 (f + e), 16.23 (c + c′ + c′′ + k) (Supplementary Materials Figure A).

FT-IR (KBr, cm^−1^): 3515 cm^−1^ (*ν*_(O–H)_), 2994 (*υ*_as_CH_3_), 2947–2945 (*υ*_as_CH_2_) (*υ*_s_CH_3_), 2868 (*υ*_s_CH_2_), 1757 (*υ*C=O), 1459 (*δ*_as_CH_3_), 1369–1383 (*δ*_s_CH_3_), 1296 (*δ*_2_CH), 1244 (*υ*_as_COC), 1189 (*υ*_s_COC) (Supplementary Material Figure B).

### 3.4. Synthesis of the bis-MPA-PLLA50-PCL50-PAM, bis-MPA-PLLA90-PCL38-PAM and bis-MPA-PLLA38-PCL90-PAM Branched Conjugates

The obtained branched copolymeric matrices—1.5 g of bis-MPA-PLLA50-PCL50, bis-MPA-PLLA90-PCL38 or bis-MPA-PLLA38-PCL90 (the *M_n_*_(SEC-MALLS)_ of the branched carriers are listed in Table 1) were first dissolved in 50 mL anhydrous THF under stirring for 30 min at room temperature. Then, succinic anhydride (the molar ratio of the copolymeric matrices to succinic anhydride was 1:1.5 for each hydroxyl group, see Table 1) and 1.5 mL of TEA as the catalyst were added to the reaction flask under reaction conditions for 24 h. The crude products (carboxyl terminal groups) were then precipitated in the cold diethyl ether twice and dried in a vacuum (the yields ranged from 82% to 86%). Subsequently, to a solution of the obtained copolymeric products with carboxyl terminal groups (0.3 g each) in methylene chloride (60 mL), *N*-hydroxysuccinimide (NHS) was added to the reaction flask under reaction conditions for 48 h (the molar ratio of the copolymeric matrices to NHS was 1:1.5 for each carboxyl group). The synthsized products (carboxyl functionalized groups) were then precipitated in the cold diethyl ether twice and dried in a vacuum (the yields ranged from 80% to 88%). After that, to the obtained functionalized copolymeric products in dioxane (60 mL), EDC and DMAP were added under argon atmosphere (the molar ratio of copolymeric products with a carboxyl functionalized group to EDC was 1:2 for each carboxyl group, and the molar ratio of EDC to DMAP was 1:1.5). The mixture was stirred at room temperature for 2 h and PAM was added (the molar ratio of copolymeric matrices with a carboxyl functionalized group to PAM was 1:1.5 for each carboxyl group). The reaction was stirred for 48 h at room temperature. The crude conjugation branched products were precipitated in a cold diethyl ether three times and dried in a vacuum. The yields of the conjugation products were 62%, 60% and 63% for bis-MPA-PLLA50-PCL50-PAM, bis-MPA-PLLA90-PCL38-PAM or bis-MPA-PLLA38-PCL90-PAM, respectively.

^1^H-NMR of bis-MPA-PLLA90-PCL38-PAM (DMSO-*d*_6_, 300 MHz, *δ*_H_, ppm): 8.22 (s, -NH- of PAM (n), 5.20 (q, -CH(CH_3_)- of PLLA (a)), 5.11 (q, -CH(CH_3_)- of PLLA (a′)), 4.13 (m, -C(O)C(CH_3_)CH_2_O of bis-MPA and (q, -CH(CH_3_), end group of PLLA (g + b′′), 3.99 (t, -CH_2_O- of PCL (f)), 3.57 (t, -CH_2_O- of PCL (f′)), 3.41 (m, -OCH_2_CH_2_O- of bis-MPA (i)), 3.24 (s, -NH-CH_2_-CH_2_- of PAM (l), 2.25 (t, -CH_2_CH_2_C(O)- of PCL (c + c′)), 2.23–2.01(m, -NH-CH_2_-CH_2_- of PAM (m) and C(O)-CH_2_-CH_2_-C(O)- of succinic anhydride (j and k), 1.62–1.22 (m, -CH_2_CH_2_C(O)- of PCL (d + d′) and (m, -CH_2_CH_2_CH_2_- of PCL (e + e′) as well as (d, -CH_3_ of PLLA (a + a′)), (d, -CH_3_, end group of PLLA (a′′), 1.19–1.01 (m, CH_3_ groups of bis-MPA (h) (Figure 2). The ^1^H-NMR spectrum of pure drug—PAM is shown in Figure C (Supplementary Materials). FT-IR (KBr, cm^−1^): 3392 cm^−1^ (*ν*_(O–H)_), 2978 (*υ*_asCH3_ and (*υ*_sCH3_), 1759 (*υ*_C=O_), 1595 *ν*(_C=O_) and *δ*(_N–H_), 1459 (*δ*_as_CH_3_), 1186 (*ν*_C–O_). 1089 (*υ*_sCOC_) (Supplementary Materials, Figure D).

### 3.5. Toxicity Assays

#### 3.5.1. Microtox Assay

A Microtox assay with the luminescent bacteria *V. fischeri* was performed with the lyophilized bacteria purchased from Modern Water (New Castle, PA, USA). The test was performed using disposable glass cuvettes. As a diluent and a control, 2% NaCl containing a 20 mM Tris buffer (pH 7.4) was used. Samples were incubated at 15 °C for 15 and 30 min and the light output of the samples was recorded with a Microtox M500 analyser (Modern Water Inc., New Castle, PA, USA). One millilitre of the extract refers to 0.9 mg of the polymer. All samples were run in triplicate.

#### 3.5.2. Spirotox Test

A Spirotox test with the ciliate protozoan *S. ambiguum* was performed according to the standard protocol [24]. The test was carried out in 24 disposable, polystyrene microplate wells. Prior to the toxicity test, the extract was neutralized with 0.1 M NaOH. Then, 0.1 mL of 0.4 M Tris (pH 7.4) was added to 10 mL of the extract. One millilitre of the extract refers to 0.99 mg of the polymer. As a diluent and a control, a Tyrod solution was used. Ten organisms were added to each of the microplate wells. The samples were incubated in the dark at 25 °C for 24 h. Following this, the test responses, i.e., the different deformations of the cell and lethal responses, were observed with the use of a dissection microscope. All samples were run in triplicate.

#### 3.5.3. The *Umu*-Test

The *umu*-test is a bioassay for evaluating the genotoxic potential of environmental samples and chemical compounds. The test organism is *S. typhimurium* TA1535/pSK1002. As a response to different types of DNA damage the umuC gene in bacterial cells is induced which is a part of the SOS system. The test strain is genetically modified—the *umuC* gene activity is linked to the synthesis of β-galactosidase while other DNA regions responsible for this enzyme synthesis were deleted. The transcription of the *umuC* gene correlates with the amount of secreted β-galactosidase. The enzyme converts colorless substrate (*ortho*-nitrophenyl-β-galactoside) into the yellow product which can be quantified colorimetrically at 420 nm. Additionally, the bacteria growth (G) is evaluated by a measurement of an optical density to determine the cytotoxicity of tested samples. The genotoxic potential of sample is presented as the Induction Ratio (IR)—the β-galactosidase activity ratio of tested sample in comparison to the negative control. Samples with IR ≥ 1.5 are considered as genotoxic. The *umu*-test was performed in 96-well microplates according to the ISO 13829 protocol with and without metabolic activation (S9 liver fraction from male Sprague-Dawley rats treated five days before the isolation with a single dose of 500 mg kg^−1^ body weight of Aroclor 1254 in soya oil) [25]. Deionized sterile water was used as a negative control, 2-aminoanthracene and 4-nitroquinoline *N*-oxide were used as positive controls. All tested copolymeric matrices were incubated in phosphate buffered saline (PBS from Gibco, Thermo Fisher Scientific, Darmstadt, Germany)—1 mg mL^−1^ for 24 h, 37 °C with shaking. Before the assay all extracts were sterilized by filtration. All samples were tested in two fold dilution series (four concentrations, the highest concentration of 0.66 mg mL^−1^). Clear PBS treated in the same way as all samples were tested as a solvent control.

### 3.6. Fabrication of the Hydroxyapatite Granules Doped with Selenite Ion

A nanocrystalline hydroxyapatite containing selenite ions (6.1 wt % of Se, the content of hydroxyapatite: 10 wt %) was prepared via standard precipitation method and previously described [18]. The obtained powder was subjected to detailed physicochemical analysis (PXRD, FT-IR, WD-XRF and TEM). In order to produce the hydroxyapatite granules, 2 g of the obtained hydroxyapatite were mixed with 25 mL 4% aqueous solution of alginate sodium. The obtained slurry was mixed carefully and then squeezed by a syringe needle into the 1.5% CaCl_2_ solution. The obtained small drops of hydroxyapatite/alginate composite was then washed several times in distilled water and dried in air at 40 °C for 24 h. The obtained material was heated in the electric oven at 600 °C for 1 h to remove organic matter and then at 1000 °C for 2 h to sinter the granules.

### 3.7. Fabrication of the Porous Material—bis-MPA-PLLA-PCL-PAM-hydroxyapatite Doped with Selenium Ion for In Vitro and Hydrolytic Biodegradation Studies

50 mg mL^−1^ synthesized copolymeric conjugates solution was prepared by dissolving a proper amount of the branched copolymeric conjugate (bis-MPA-PLLA50-PCL50-PAM, bis-MPA-PLLA90-PCL38-PAM or bis-MPA-PLLA38-PCL90-PAM) in 10 mL of methylene chloride. 10 mg of the hydroxyapatite granules doped with selenium ions were soaked in 2 mL of the copolymeric conjugate solution at 37 °C for 24 h to load the branched PAM conjugate into the hydroxyapatite porous material. After the solution was filtred, the remained material was gently washed with distilled water to remove any physically absorbed conjugate solution. For the hydrolytic biodegradation, the porous materials composed of the branched copolymeric carrier coated hydroxyapatite granules doped with selenite ions have been manufactured in the same way as for release studies.

### 3.8. In Vitro PAM Release Studies form the Synthesized Conjugates Coated Hydroxyapatite Porous Granules Doped with Selenite Ions

The in vitro release study of drug (PAM) was performed to measure the concentration of PAM released at pH 7.4 ± 0.05 (0.1 M phosphate buffer solution) for 7 days. All experiments were carried out in triplicate; a total of 10 mg each of dried hydroxyapatite porous materials coated with the synthesized conjugates (c = 50 mg mL^−1^) were immersed in 2 mL buffer solution and incubated at 37 °C with continuous orbital rotation at 50 cycles/min. At predetermined time intervals, samples were withdrawn from the release medium and then analysed by means of HPLC CAD.

### 3.9. Hydrolytic Degradation of the Synthesized Copolymeric Matrices Coated Hydroxyapatite Porous Granules Doped with Selenite Ion

The hydrolytic degradation of the manufactured porous material (porous hydroxyapatite granules doped with selenite ions and coated with branched polymeric matrices (bis-MPA-PLLA50-PCL50; bis-MPA-PLLA90-PCL38 and bis-MPA-PLLA38-PCL90) was performed in a 10 mL phosphate buffer solution (pH 7.4 ± 0.05) at 37 °C for 7 days. Following hydrolysis, the composite material samples were washed intensively with distilled water to remove any residual buffer solution, followed by drying under reduced pressure for four days. The degradation rates were estimated by weight loss (WL, (%)), calculated with the following equation:

WL (%) = 100 × (W_0_ – W_t_)/W_0_

where, W_0_ = initial weight and W_t_ = weight after degradation.

## 4. Conclusions

New multifunctional composites, composed of biodegradable, branched copolymer-PAM conjugate coated porous hydroxyapatite granules doped with selenite ions were effectively fabricated in this study. Bulk ROP of CL and LA, initiated by bis-MPA molecule was carried out in order to obtain branched copolymeric matrices. The structure and physicochemical properties of the synthesized copolymers was confirmed by ^1^H-, ^13^C-NMR, FT-IR and SEC-MALLS methods. The resulting matrices were also subjected to cyto- and genotoxicity assays using bacterial luminescence, protozoan and *S. typhimurium* TA1535 assays. The *in vitro* release studies showed the dependence of the copolymeric carrier composition on the PAM release profile; the most amount of PAM (66%) was released from the bis-MPA-PLLA90-PKL38-PAM copolymeric conjugate, whereas only 31% of PAM was released from bis-MPA-PLLA38-PKL90-PAM sample, however in this case it was in the most uniform and controlled manner. Therefore, the fabricated multifunctional composite could allow to increase PAM therapeutic benefit since its pharmacokinetics might be improved by the hydrolysis of the newly formed amide bond. It is also important to note that a potential cancer therapy can be assisted by the presence of selenite ions in the manufactured composite.

## Figures and Tables

**Table 1:** Copolymerization of LLA and CL initiated by bis-MPA. Characterization of the synthesized branched copolymers.

Entry	[I]/[LLA]/[CL]	Yield (%)	*DP* ^a^	*DS ^a^*	*M*_n(SEC-MALLS)_^b^	*M*_w_/*M*_n_^b^
bis-MPA-PLLA64-PCL64	1/64/64	82	25	13	21000	1.5
bis-MPA-PLLA90-PCL38	1/90/38	85	22	10	18800	1.6
bis-MPA-PLLA38-PCL90	1/38/90	97	19	11	19500	1.5

*Reaction conditions*: argon atmosphere, reactions temperature—135 °C (for homopolymerization), 110 °C (for copolymerization); l,l-lactide (LLA), ε-caprolactone (CL), I = initiator—bis-MPA; ^a^ *DP*—the average degree of polymerization determined by ^1^H-NMR analysis, calculated based on the area ratio of the terminal methylene protons of PCL (3.55 ppm) or methine proton of PLLA (4.12 ppm) to the internal methylene proton of PCL (3.98 ppm) or methine proton of PLLA (5.13 ppm); *^a^ DS*—the average degree of substitution determined by ^1^H-NMR analysis; calculated based on the signal areas or the methylene protons of PCL (3.55 ppm) or methine proton of PLLA (4.12 ppm) and methylene protons of bis-MPA (1.19–1.01 ppm); ^b^ *M*_n(SEC-MALLS)_ and *M*_w_/*M*_n_ determined using SEC-MALLS instrument.

**Table 2:** The cytotoxicity results of the synthesized branched copolymeric matrices.

Entry	Microtox 15 min-PE ^1^ 0.9 mg mL^−1^	Microtox 30 min-PE ^1^ 0.9 mg mL^−1^	Spirotox 24 h-PE ^1^
bis-MPA-PLLA64-PCL64	10 ± 1	11 ± 2	0
bis-MPA-PLLA90-PCL38	14 ± 2	18 ± 1	0
bis-MPA-PLLA38-PCL90	4 ± 2	5 ± 1	0

^1^ Percent of toxic effect.

**Table 3:** The results of the *umu*-test for the highest concentrations of the tested branched copolymeric matrices extracts (0.66 mg mL^−1^).

Entry	−S9		+S9	
	G ± SD	IR ± SD	G ± SD	IR ± SD
bis-MPA-PLLA64-PCL64	1.09 ± 0.03	0.81 ± 0.09	1.18 ± 0.04	0.89 ± 0.09
bis-MPA-PLLA90-PCL38	1.06 ± 0.05	0.88 ± 0.07	1.16 ± 0.02	0.95 ± 0.03
bis-MPA-PLLA38-PCL90	0.98 ± 0.05	0.96 ± 0.02	1.15 ± 0.03	0.96 ± 0.04

## Supplementary Materials

The following are available online, Figure A: ^13^C-NMR spectra of bis-MPA-PLLA38-PCL90, Figure B: FT-IR spectra of bis-MPA-PLLA38-PCL90, Figure C: ^1^H-NMR of disodium pamidronate, Figure D: FT-IR spectra of bis-MPA-PLLA90-PCL38-PAM.
