# Supplementary figures and images for: Pamidronate-Conjugated Biodegradable Branched Copolyester Carriers: Synthesis and Characterization
Source: Molecules. 2017 Jun 26;22(7):1063. doi: 10.3390/molecules22071063 (PMC6151985; doi:10.3390/molecules22071063)

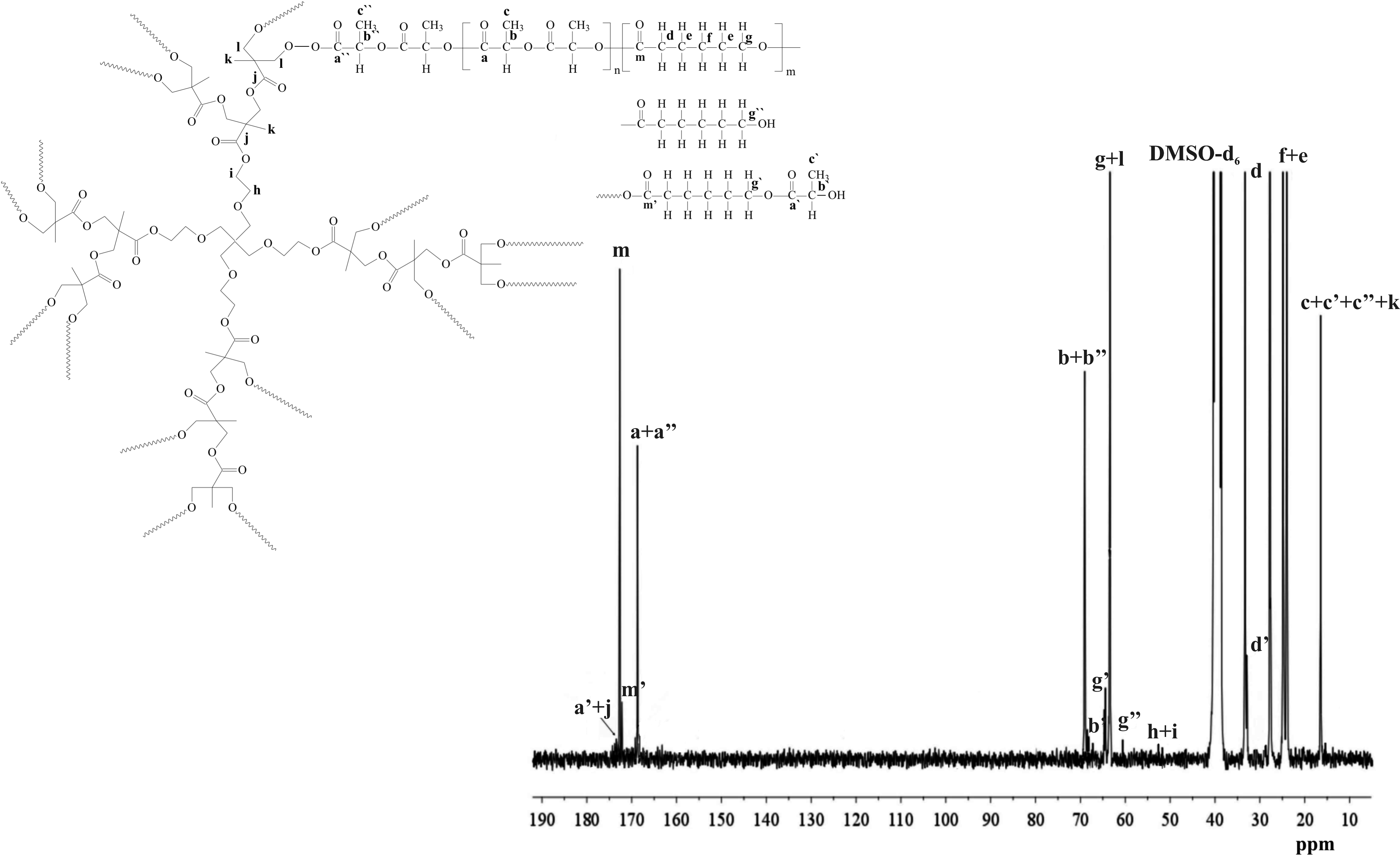

Supplement: Supplementary file 1 [file molecules-22-01063-s001.zip › molecules-198541-supplementary/Supp. Info Figure A.tif]

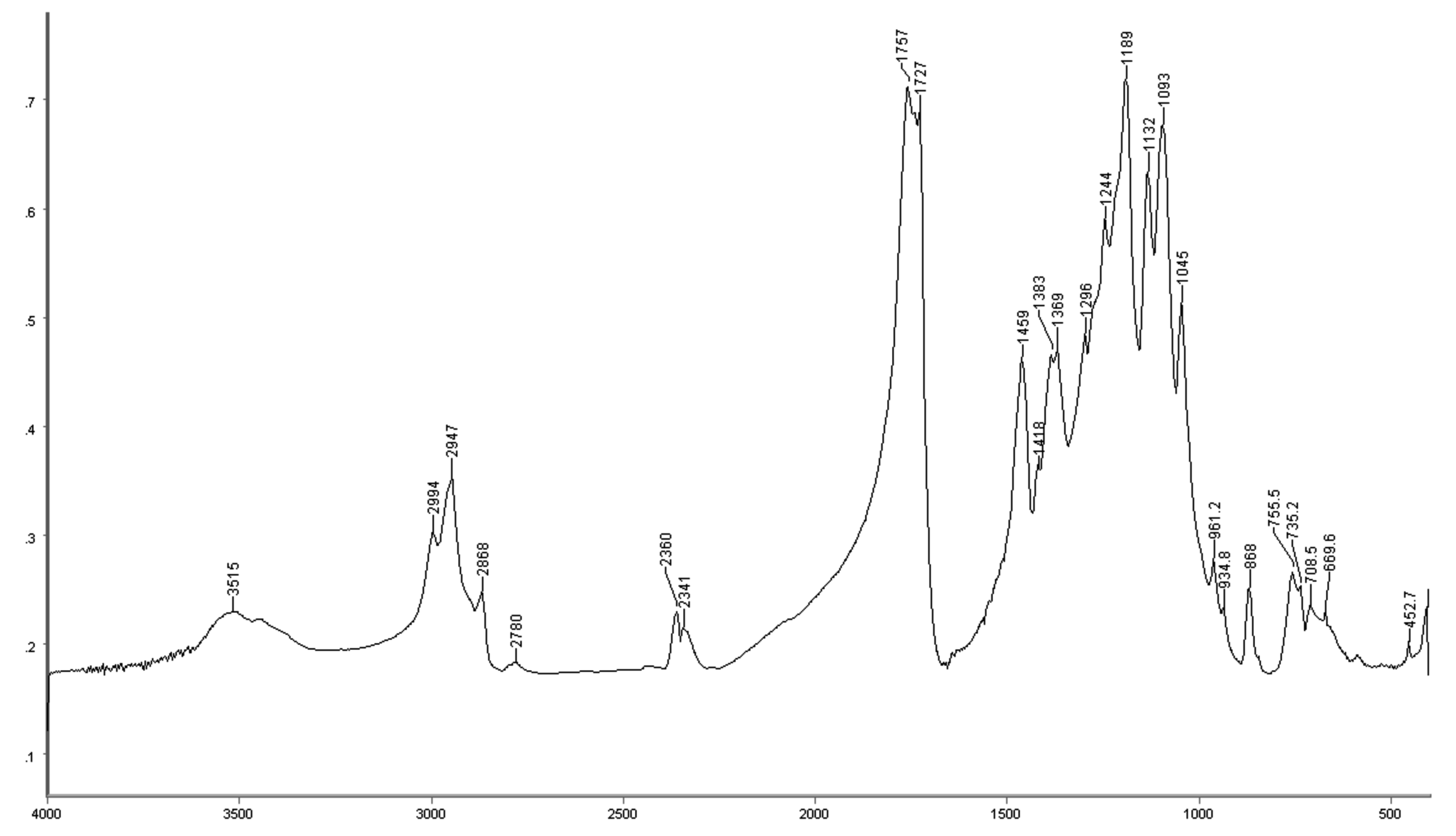

Supplement: Supplementary file 1 [file molecules-22-01063-s001.zip › molecules-198541-supplementary/Supp. Info Figure B.tif]

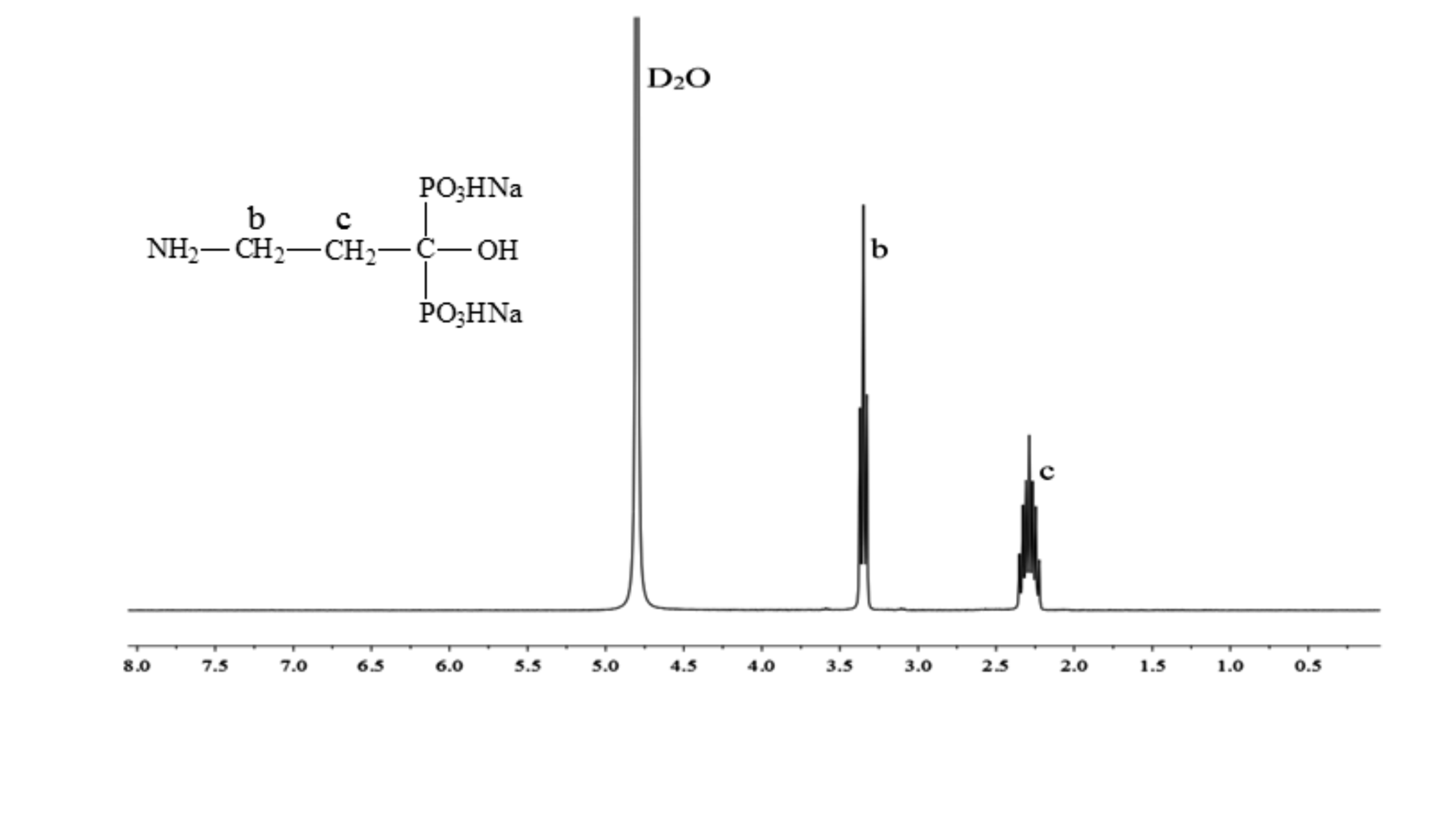

Supplement: Supplementary file 1 [file molecules-22-01063-s001.zip › molecules-198541-supplementary/Supp. Info Figure C.tif]

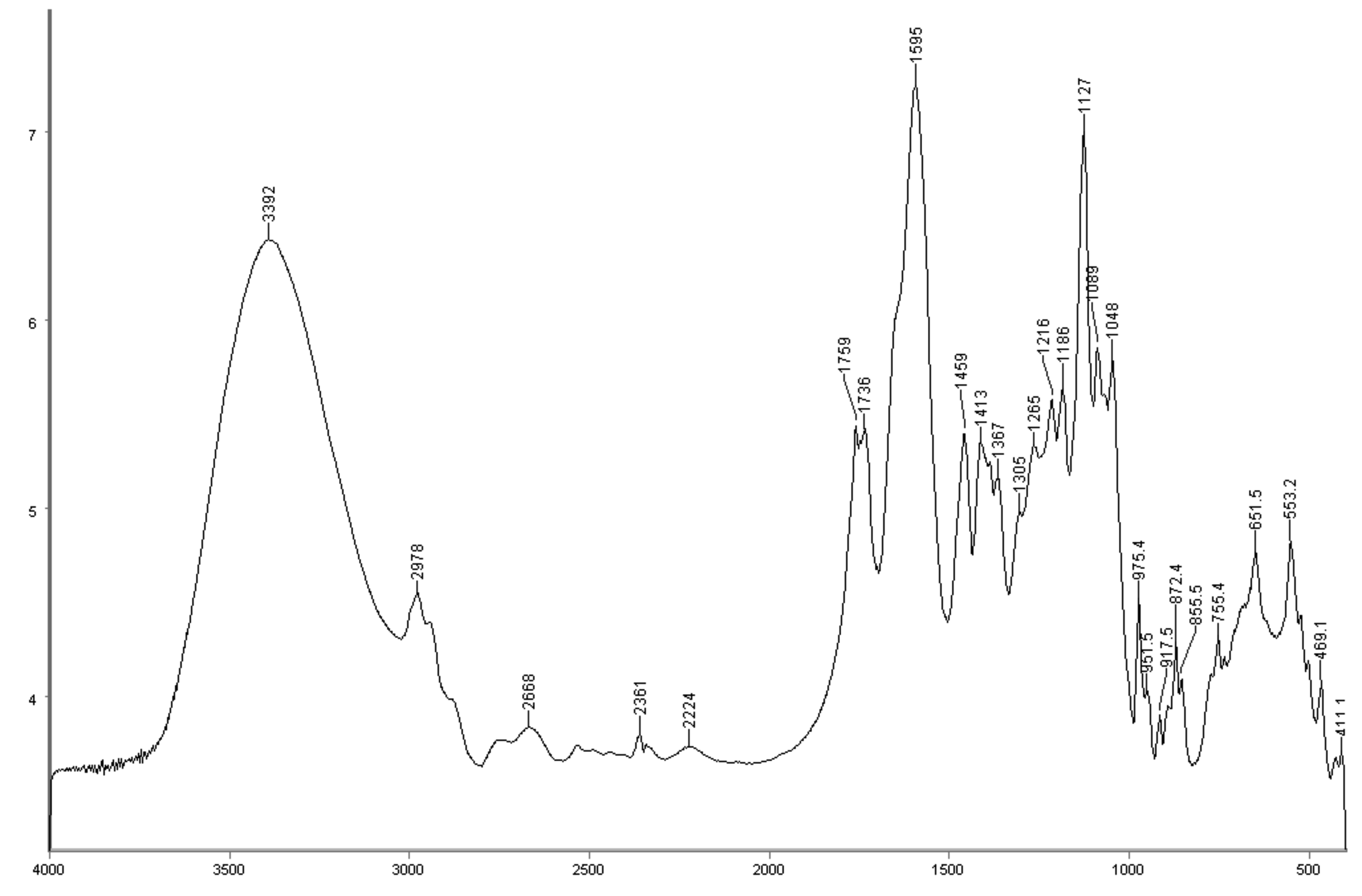

Supplement: Supplementary file 1 [file molecules-22-01063-s001.zip › molecules-198541-supplementary/Supp. Info Figure D.tif]
